# Quantum anti-Zeno effect without wave function reduction

**DOI:** 10.1038/srep01752

**Published:** 2013-05-08

**Authors:** Qing Ai, Dazhi Xu, Su Yi, A. G. Kofman, C. P. Sun, Franco Nori

**Affiliations:** 1 CEMS, RIKEN, Saitama 351-0198, Japan; 2 Institute of Theoretical Physics, Chinese Academy of Sciences, Beijing 100190, China; 3 Physics Department, The University of Michigan, Ann Arbor, Michigan 48109-1040, USA; 4 Beijing Computational Science Research Center, Beijing 100084, China; 5 Department of Physics, Korea University, Seoul 136-713, Korea

## Abstract

We study the measurement-induced enhancement of the spontaneous decay for a two-level subsystem, where measurements are treated as couplings between the excited state and an auxiliary state rather than the von Neumann's wave function reduction. The photon radiated in a fast decay of the atom, from the auxiliary state to the excited state, triggers a quasi-measurement, as opposed to a projection measurement. Our use of the term “quasi-measurement” refers to a “coupling-based measurement”. Such frequent quasi-measurements result in an exponential decay of the survival probability of atomic initial state with a photon emission following each quasi-measurement. Our calculations show that the effective decay rate is of the same form as the one based on projection measurements. The survival probability of the atomic initial state obtained by tracing over all the photon states is equivalent to that of the atomic initial state with a photon emission following each quasi-measurement.

In the quantum Zeno effect (QZE) (see, e.g., Refs. 1,2,3,4,5,6) frequent measurements inhibit atomic transitions for a closed system. In the quantum anti-Zeno effect (QAZE), atomic decays can be accelerated by frequent measurements, when the observed atom also interacts with a heat bath with some spectral distribution^7^,^8^,^9^,^10^,^11^,^12^,^13^,^14^,^15^. This QAZE has been extensively studied for various cases, such as the QAZE without the rotating-wave approximation^10^,^11^,^15^,^16^ and in an artificial bath^12^. The conventional explorations for the QAZE as well as the QZE need to invoke the von Neumann's wave function collapse^17^ for quantum measurements, namely the projection measurement postulate. Thus, the QAZE seems to depend on a particular quantum mechanical interpretation specified by this collapse postulate.

However, even though the collapse postulate has been extensively used in the past, some researchers do not believe it is necessary for quantum mechanics. There exist other interpretations, such as the ensemble interpretation^18^. In this sense, it is necessary to develop a quantum-mechanical-interpretation-independent approach to the QAZE.

To this end, we draw lessons from the dynamic explanations of the QZE^19^,^20^,^21^,^22^,^23^. After the QZE was proposed by Misra and Sudarshan^6^, it was recognized^24^ that the QZE could be mimicked by strong couplings to an external agent, which carried out a coupling-based detection. Then, an experiment^25^ observing the QZE was explained^21^ in such a dynamic fashion. Therein, all the phenomena were only described by the unitary evolution governed by the Schrödinger equation for the whole system. Later on, to further develop this dynamic interpretation of the QZE, Pascazio *et al.*^26^ and Sun *et al.*^27^,^28^ explicitly used the decoherence model of quantum measurement, where the couplings to the apparatus only decohered the phases of the system rather than changed the system's energy. This measurement model is essentially a non-demolition measurement^29^,^30^,^31^,^32^.

Following these dynamic approaches for the QZE, we now develop a quantum dynamic theory for the QAZE without reference to projection measurements or the collapse postulate. To illustrate our main idea, we use an example: a two-level subsystem coupled to an auxiliary state to form a cascade configuration. Due to the couplings to the reservoir, the excited state spontaneously decays to the ground state. After a short interval, the remaining population of the excited state is coherently pumped into the auxiliary state by a strong laser. Then, it returns to the excited state by a fast spontaneous decay and a photon is emitted simultaneously. At this stage, a quasi-measurement is realized. Here, the term *quasi-measurement* refers to a *coupling-based measurement* in contrast to the usual projection measurement. The correlation of the atomic initial state and the orthogonal states with two orthogonal states of the environment is produced in such a process. We call it quasi-measurement since it can be viewed as the first (unitary) stage of the measurement process. Similar to the conventional approach, based on the collapse postulate, the effective decay rate of the survival probability with one photon emitted following each pulse in the presence of such quasi-measurements is given by the overlap integral of the measurement-induced level-broadening function and the interacting spectral distribution. As different photon states may not be distinguished in a realistic experiment, the survival probability of the atomic initial state after *n* repetitive quasi-measurements, which can be obtained by tracing over all the photon states, can be taken into consideration. The contributions from photon states with less than *n* emitted photons correspond to repopulation of the initial state due to return of the excitation from the reservoir. We calculate these contributions and show that they are small and can be omitted in the first approximation under a weak coupling. Thus, the result for the projection measurements is recovered with the quasi-measurements.

## Results

### Model setup

**Figure 1:**
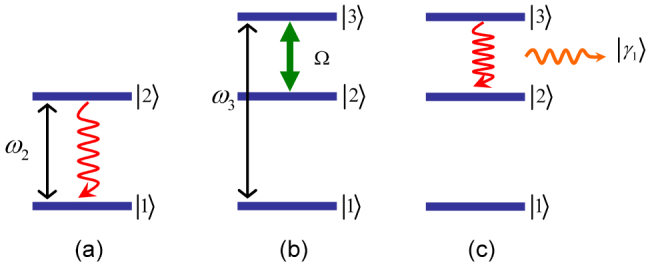
Energy level diagram for the three processes considered here: (a) the spontaneous decay from the excited state |2〉 to the ground state |1〉, (b) a coherent transition with Rabi frequency Ω between |2〉 and the auxiliary state |3〉 by laser pumping, and (c) a fast spontaneous decay from |3〉 to |2〉 with a photon emitted in |γ_1_〉. Here, the eigenenergies for the excited state and the auxiliary state are *ω*_2_ and *ω*_3_, respectively.

We consider the QAZE for a three-level atom with the cascade configuration depicted in Fig. 1(b, c). We mainly focus on the QAZE concerning a subsystem with the ground state |1〉 and the excited state |2〉 [see Fig. 1(a)]. Since these two levels are coupled to a reservoir, there would be natural spontaneous decay from |2〉 to |1〉 if the subsystem were not coupled to other dynamic agents. In this process with duration *τ*, the total system is governed by the Hamiltonian 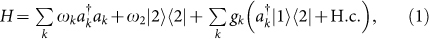
where 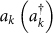 is the annihilation (creation) operator for the reservoir's *k*th mode with frequency *ω_k_*, *ω*_2_ the eigenenergy for the excited state |2〉, and *g_k_* the coupling constant between the *k*th mode and the transition between |1〉 and |2〉, which is assumed to be real for simplicity. We assume *ω*_1_ = 0. Notice that we have applied the rotating-wave approximation^33^ to the above Hamiltonian (1).

**Figure 2:**
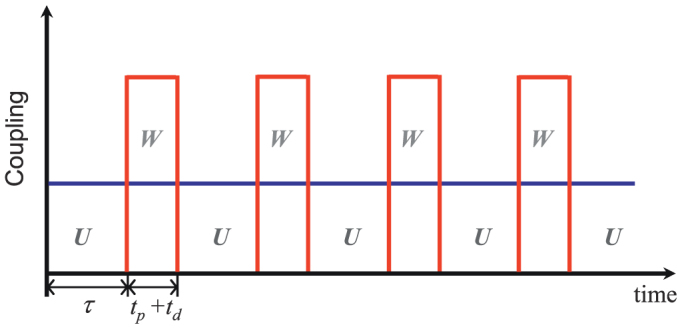
The pulse sequence for demonstrating the QAZE by a quasi-measurement (i.e., avoiding projection measurements). Here, *U* stands for the spontaneous decay from |2〉 to |1〉. Also, *W* is a quasi-measurement which is alternatively present and absent for a duration *t_p_* + *t_d_* and *τ*, respectively.

In order to perform a quasi-measurement, we avoid the collapse postulate, as also done, e.g., in Refs. 19, 20, 21, 24, where the quasi-measurement involved coherently coupling the measured state to an external agent, e.g., an additional energy level |3〉. In this sense, a quasi-measurement is the first (unitary) stage of the measurement process, providing an entanglement between the system and the apparatus. A quantum measurement in this approach is implemented by an alternative coupling^18^,^19^,^20^,^21^,^23^,^25^ lasting for *t_p_* between |2〉 and |3〉 with eigenenergy *ω*_3_, which is described by 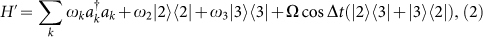
where Ω is the Rabi frequency between |2〉 and |3〉. Hereafter, we focus on the resonance case, i.e., 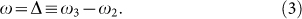
When the resonant coupling laser is applied between |2〉 and |3〉, we can disregard the spontaneous decay between the auxiliary state |3〉 and the excited state |2〉 for a very strong laser, i.e., 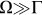, with a short pulse duration 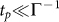, where Γ is the decay rate from |3〉 to |2〉. We assume that each laser pulse is a *π*-pulse, which transfers the population of state |2〉 to |3〉. Then, when the coupling laser is turned off, the population of the state |3〉 will quickly return to |2〉, with a photon *γ* produced by the spontaneous decay, i.e., 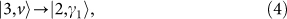
where |3, *v*〉 = |3〉 |*v*〉 is the product state of the atomic auxiliary state |3〉 and the vacuum |*v*〉 for the reservoir, |*γ_n_*〉 denotes the state with *n* photons in the *γ* mode. At this stage, the quasi-measurement is completed. Then, the subsystem alternatively evolves freely and is “measured” through laser pumping. The time sequence for the entire course is schematically shown in Fig. 2.

Here, we assume the duration 1/Γ for the fast spontaneous decay from the auxiliary state |3〉 to the excited state |2〉 to be much smaller than the one for the spontaneous decay from |2〉 to |1〉 and than the interval between the pulses, i.e., 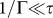. In this case, we can omit the dynamic evolution between |2〉 and |1〉 induced by the finite couplings to the reservoir when the fast spontaneous decay from the auxiliary state |3〉 to the excited state |2〉 occurs. On the other hand, notice that we treat the photons emitted by the decay of the atom from |2〉 to |1〉 and the photons emitted by the decay from |3〉 to |2〉 separately. Here, we assume that the level spacing between |2〉 and |1〉 is largely detuned from the one between |3〉 and |2〉. Then the frequency range of the photons emitted in the decay from |3〉 to |2〉 does not overlap the one of the photons emitted in the decay from |2〉 to |1〉. In this case, we can consider the spontaneous decay for the two transitions as occurring to different reservoirs and thus treat the two different kinds of photons separately. Below we call the reservoirs corresponding to the transitions between |1〉 and |2〉, and between |2〉 and |3〉, as reservoirs 1 and 2, respectively.

### Dynamical approach to the anti-Zeno effect

Previously, we described a dynamical approach to study the QAZE. We emphasize that in our approach there is no wave-function-reduction postulate involved, and the unitary evolution of both the two-level subsystem and the measuring apparatus is depicted by means of the Schrödinger equation. Let us first describe the two basic processes *U* and *W* schematically illustrated in Fig. 2.

For the spontaneous decay between the excited state and the ground state, governed by the Hamiltonian (1), we assume the wave function of the total system to be a superposition of two kinds of single-excitation states, i.e., 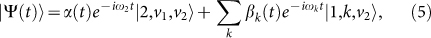
where |2, *v*_1_, *v*_2_〉 = |2〉 |*v*_1_〉 |*v*_2_〉 is the product state of the atomic excited state |2〉 and the vacuum states |*v*_1_〉 and |*v*_2_〉 for the reservoirs 1 and 2, respectively |1, *k*, *v*_2_〉 = |1〉 |*k*〉 |*v*_2_〉, with |*k*〉 being the single-excitation state in the *k*th mode of the reservoir corresponding to the transition |1〉–|2〉, and *ω_k_* is the *k*th-mode frequency. It follows from the Schrödinger equation *i*∂*_t_*|Ψ(*t*)〉 = *H* |Ψ(*t*)〉 that the coefficients *α*(*t*) and *β_k_*(*t*) in equation (5) satisfy 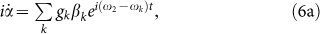

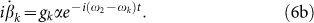


Under the short-time approximation, the solutions to the above equations for *t* ≥ *t*_0_ become 
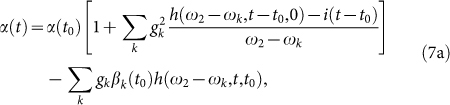

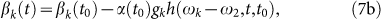
where 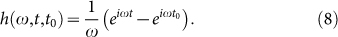
The detailed calculations are presented in Methods section.

In the quasi-measurement process, a strong laser field is applied to induce the transition between the excited state |2〉 and the auxiliary state |3〉 [see Fig. 1(b)]. With a unitary transformation 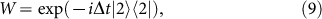
the transformed wave function |Ψ′(*t*)〉 ≡ *W* |Ψ(*t*)〉 is governed by the effective Hamiltonian *H*_eff_ ≡ *WH′W*^†^ – *iW*∂*_t_W*^†^, which reads 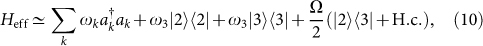
where we have dropped the fast-oscillating terms including the factors exp(±*i*2Δ*t*).

Now we assume the transformed wave function to be 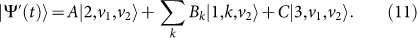
Then the original wave function |Ψ(*t*)〉 = *W*^−1^ |Ψ′(*t*)〉 can be written as 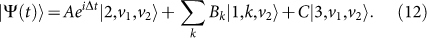
According to the Schrödinger equation for the transformed wave function *i*∂*_t_*|Ψ′(*t*)〉 = *H*_eff_ |Ψ′(*t*)〉, we obtain the following system of differential equations 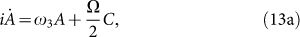

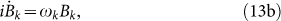

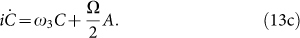
The solutions are given by 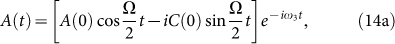

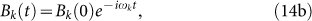

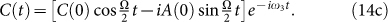
Applying a *π*-pulse, i.e., a laser with duration 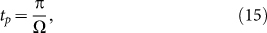
drives the system to evolve into the state 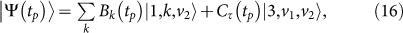
where the coefficients 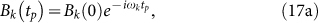


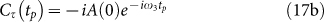
can be obtained from equation (14). Here, we have assumed there is no initial population in the auxiliary state, namely *C*(0) = 0, and thus *A*(*t_p_*) = 0. Afterwards, by means of a fast spontaneous decay, the state |3, *v*_1_, *v*_2_〉 decays into |2, *v*_1_, *γ*_1_〉 [see Fig. 1(c)]. Therefore, a quasi-measurement is finished.

Here, we will explicitly describe the complete process including the free evolution by *U* and the quasi-measurement by *W*. The total system is initially prepared in the excited state with the reservoirs in the vacuum: |Ψ(0)〉 = |2, *v*_1_, *v*_2_〉. Then, according to equations (5) and (7), after a free evolution with period *τ*, the state evolves into^34^

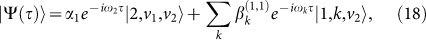
where 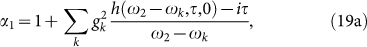

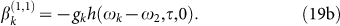
Applying a strong laser forces the system to evolve into [cf. equation (17b)] 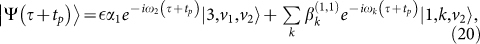
where 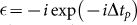. Later, through a fast spontaneous decay, the total system becomes 
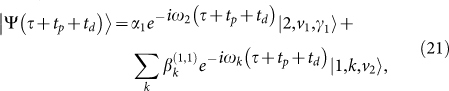
where *t_d_* ~ Γ^−1^ and the phase factor 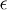 is included in the wave function |*γ*_1_〉. At this stage, the first cycle is accomplished. The survival probability amplitude of the state |2〉 after one quasi-measurement is *α*_1_. Hereafter, for the sake of simplicity, we will label |Ψ(*nτ* + (*n* − 1)*t_p_* + (*n* − 1)*t_d_*)〉, |Ψ(*nτ* + *nt_p_* + (*n* − 1)*t_d_*)〉, and |Ψ(*n* (*τ* + *t_p_* + *t_d_*))〉 as |Ψ*_n_*(1)〉, |Ψ*_n_*(2)〉, and |Ψ*_n_*(3)〉, respectively. In other words, |Ψ*_n_*(*j*)〉 denotes the state after *j*th procedure in the *n*th cycle for *n* = 1, 2, … and *j* = 1, 2, 3.

For the second cycle, after the free evolution, the total system is in the state 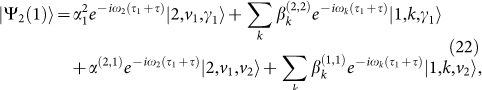
where *τ_n_* = *n*(*τ* + *t_p_* + *t_d_*), 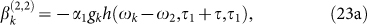

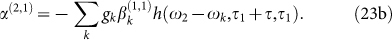


The coefficients 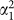 and 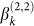 are determined by *α*(*τ*_1_ + *τ*) and *β_k_*(*τ*_1_ + *τ*), respectively, in equation (7) with initial conditions at *t*_0_ = *τ*_1_

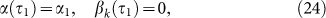
whereas *α*^(2,1)^ and 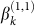 are determined by *α*(*τ*_1_ + *τ*) and *β_k_*(*τ*_1_ + *τ*), respectively, with initial conditions 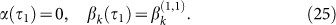
After another *π*-pulse, 
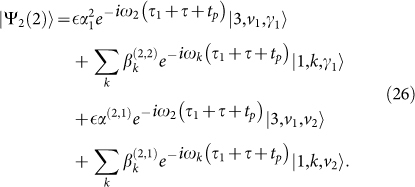
Afterwards, by means of a fast spontaneous decay it becomes 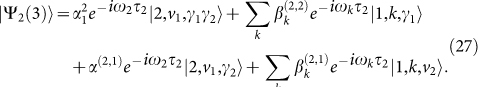
Here the phase factors 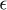 are included in the photon states, |2, *v*_1_, *γ*_1_*γ*_2_〉 = |2〉|*v*_1_〉|*γ*_1_*γ*_2_〉, |*γ_i_γ_j_* …〉 is the state with the photons *γ_i_*, *γ_j_*, …, and *γ_i_* is the photon emitted in the *i*th cycle.

Thus, the survival probability of the state |2〉 with photon emissions following both pulses is |*α*_1_|^4^. Here, we point out that this is different from the survival probability of the atomic initial state, which has an additional contribution from |2, *v*_1_, *γ*_2_〉. In this dynamic approach for the QAZE, once a photon in the *γ* mode is emitted right after a pulse, a quasi-measurement is finished. This means that the system is in the initial state before the quasi-measurement and still remains in its initial state after the quasi-measurement. For the case with two quasi-measurements, *α*^(2,1)^ corresponds to such a probability amplitude which decays to the ground state before the first quasi-measurement and returns to the excited state before the second quasi-measurement.

**Figure 3:**
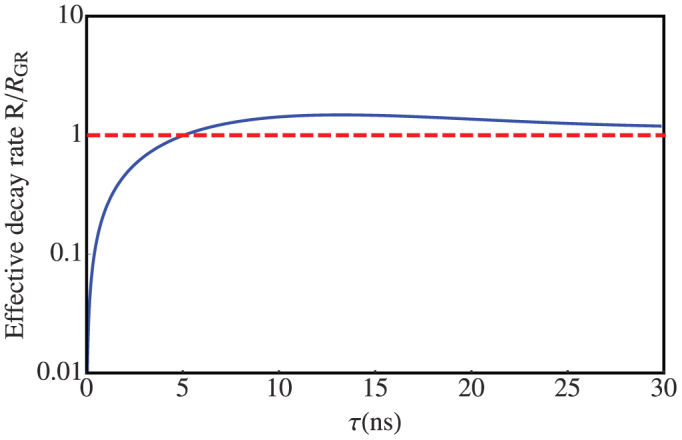
The effective decay rate *R*(*τ*) versus the quasi-measurement interval *τ* for a Lorentzian interacting spectrum with *δ* = 10^8^ rad/s, *ω*_2_ = 10^12^ rad/s, *ν* − *ω*_2_ = 2 × 10^8^ rad/s, and *R*_GR_ = 10^7^ rad/s. The red dashed line displays the golden-rule decay rate.

Judging from the analysis made in the above calculation, we may safely arrive at the conclusion that the survival probability amplitude of the state |2〉 with photon emissions following *n* pulses is 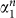. It is straightforward to calculate the survival probability as 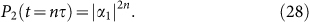
As a result, we observe an exponential decay of the survival probability of the atomic initial state with photon emission following each pulse, i.e., 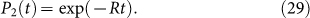
Here, the effective decay rate is^8^

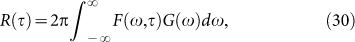
where the interaction spectral distribution is 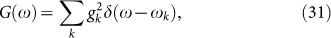
and the measurement-induced level-broadening function is given by 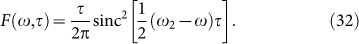
We now perform the numerical simulation for a Lorentzian interacting spectral distribution 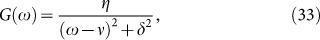
where *ν* and *δ* are the center and the width of the spectrum respectively, *η* the coupling strength. For typical neutral atoms, the spontaneous decay rates are of the orders of 10^7^–10^9^ rad/s, e.g., 5.51 × 10^7^ rad/s for 6 ^3^*P*_1_-6 ^1^*S*_0_ of mercury^35^ and 1.26 × 10^9^ rad/s for 4*s*4*p*^1^*P*_1_-4*s*^21^*S*_0_ of calcium^36^. Here, we choose the following parameters *δ* = 10^8^ rad/s, *ω*_2_ = 10^12^ rad/s, *ν* = *ω*_2_+2*δ*, *η* = 10^24^/(4*π*) rad^3^/s^3^ and Γ = 10^9^ rad/s. It is worth noticing that the detuning between the center of interacting spectrum and transition frequency between the excited and ground states is 2*δ*. We remark that in collaboration with other parameters the detuning plays the center role in the existence of the QAZE. Therefore, the golden-rule decay rate is straightforwardly obtained as *R*_GR_ = 10^7^ rad/s. As shown in Fig. 3, both the QAZE and QZE can be observed if the quasi-measurement interval *τ* is suitable, as predicted by the projection measurement^8^. And the above analysis is reasonable since the transition time of the QAZE to QZE 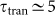 fulfills the requirement 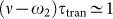, which represents the border between the regions of QAZE and QZE, i.e., 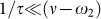 and 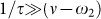, cf. Ref. 8, respectively. Additionally, because the atom-bath coupling is in the weak-coupling regime, e.g., sufficiently-large *δ*, as will be shown in the next section, the contribution from the survival probabilities with less than n photons emitted can be omitted.

### Effect of repopulation

In the previous deduction, we neglected measurement-assisted return of the excitation from the reservoir, which repopulates the initial level. Such an assumption is usually done in the treatments of the quantum Zeno and anti-Zeno effects on exponential spontaneous decay^8^,^34^. In other words, it is usually assumed that after each (quasi-)measurement the decay into the *empty* reservoir is resumed. However, strictly speaking, the reservoir is not empty, and the emitted photon can be reabsorbed. Let us consider this effect in more detail.

In the intervals between (quasi-)measurements, the state of the system, comprised of the transition 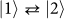 and reservoir 1 (call it system *S*), is entangled with the state of reservoir 2, so that the total wave function is a superposition of products comprised of a state of system *S* and a state corresponding to a certain set of photons emitted to reservoir 2. States of system *S* multiplied by different states of reservoir 2 undergo unitary evolution independently of each other. After a quasi-measurement, each state of the form |2〉|*ψ*_1_〉|*ψ*_2_〉 in |Ψ*_n_*(1)〉, where |*ψ_j_*〉 is a state of reservoir *j* (*j* = 1, 2), becomes 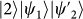 in |Ψ*_n_*(3)〉, where 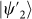 is orthogonal to all states of reservoir 2 in |Ψ*_n_*(1)〉; in contrast, all states of the form |1〉|*ψ*_1_〉|*ψ*_2_〉 remain unchanged. This destroys the coherence between the initial and final states in spontaneous decay. As a result, unitary evolutions of different components of the wave function in the interval (*τ_n_*, *τ_n_*+*τ*) are of two types: (a) spontaneous decay into the empty reservoir and (b) return of the excitation from the reservoir to the empty level |2〉. The two types of evolution have initial conditions similar to equations (24) and (25), respectively.

To estimate the effect of repopulation on the evolution of the excited state, we consider sufficiently short times, where 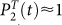. In this time interval, it is sufficient to consider the corrections to equation (28) due to the cases where the excitation only once leaves the atom and then returns back, contributions due to the cases where the excitation leaves the atom two and more times being of higher orders of smallness. Taking into account the above corrections due to repopulation, we obtain that the survival probability of the atomic initial state reads 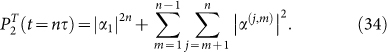
Here *α*^(^*^j,m^*^)^ is the amplitude of the process in which the excitation leaves the atom in the *m*th cycle and returns to the initial state in the *j*th cycle.

As shown in Methods section, 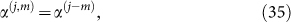
where 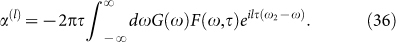
As follows from equation (35), equation (34) can be recast as 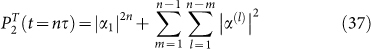
or, equivalently, 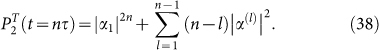


Equation (36) implies that *α*^(*l*)^ tends to zero when *l* tends to infinity, the characteristic decay value of *l* being 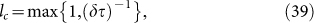
where *δ* is the width of *G*(*ω*). [Here it is assumed, for simplicity, that *G*(*ω*) is a smooth function, the shape of which is characterized by a single width *δ*, such as a Lorentzian or a Gaussian distribution.

For 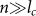, we can obtain from equation (37) with the account of equation (29) that 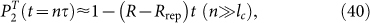
where the repopulation rate 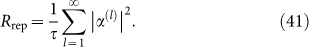
Equation (40) shows that repopulation somewhat reduces the decay rate *R*.

Consider the conditions under which the effect of repopulation is negligibly small, i.e., 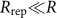. It is easy to see that 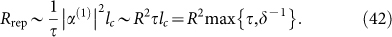
Here to obtain the first, second, and third relations, we used equations (41), (36), and (39), respectively. Thus, the inequality 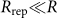 holds, when the following conditions are valid simultaneously, 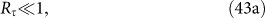

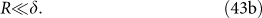
Condition (43a) is inherent in the present formalism, since it is assumed here that the interval between measurements is so small that the change of the initial state is small during the time *τ*. Condition (43b) implies that the coupling to the reservoir is sufficiently weak, which holds when the function *G*(*ω*) is sufficiently broad and smooth. This condition is very close to the condition of an exponential decay^37^, 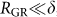, where *R*_GR_ is the golden-rule (unperturbed) decay rate.

Under conditions (43), the result (40) can be extended to all times. Consider some time *t*′ satisfying the condition 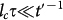. Then it is easy to show that 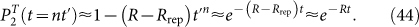
The last expression in equation (44) shows that the effect of repopulation on Zeno and anti-Zeno dynamics can be neglected under conditions (43).

The situation is significantly different, when *δ* is very small or vanishes. In this case of a strong coupling, the free evolution of the initial state is not exponential, but rather oscillatory, involving depopulations and repopulations of the initial state^37^. Then condition (43b) is violated, and the approximate result (40) also does not hold, since the inequality 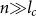 cannot be fulfilled in the short-time region. Now Zeno and anti-Zeno dynamics significantly depend on repopulation.

As a simple example, consider the extreme case *δ* = 0, i.e., 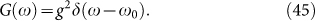
Then equation (36) becomes 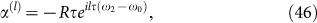
where, in view of equation (30), 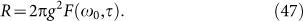
For 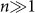, equation (38) with the account of equations (29) and (46) yields 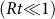


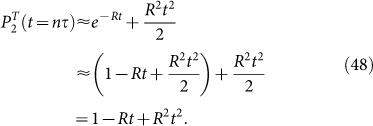
Equation (48) shows that now repopulation modifies significantly, at least, the second-order term in the exponential decay (29). Similarly, one can expect that higher-order terms are also modified by repopulation, though this modification is out of the scope of the present theory. The second- and higher-order terms become important for 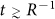, i.e., now repopulation significantly affects the quantum Zeno/anti-Zeno dynamics, at least, for sufficiently long times.

The QZE on resonant Rabi oscillations was studied for discrete measurements in Refs. 19, 20, 22, 23, 25, 38. Now the “reservoir” is described by equation (45) with *ω*_0_ = *ω*_2_, whereas 2*g*is the Rabi-oscillation frequency. In this case, for sufficiently short *τ* satisfying equation (43a), the Zeno dynamics for all times has the form 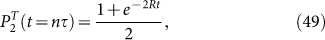
where, in accordance with equation (47), 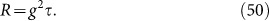
A similar result also holds, when discrete measurements are substituted by weak continuous measurements^39^. Up to the second order in time, equation (49) coincides with equation (48), whereas for 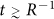, equation (49) significantly differs from equation (29), in agreement with the above discussion. The QZE and QAZE with the account of repopulation in a case when equation (45) does not hold were studied in Ref. 40.

## Discussion

In this paper, we investigated the QAZE for a two-level subsystem embedded in a three-level atom. Instead of considering projection measurements, we studied quasi-measurements by pumping the population of the excited state to an auxiliary state. Since the pumped population returns to the excited state by a fast spontaneous decay, the complete process of the quasi-measurement is finished. Along with the fast spontaneous decay, there is a photon emitted in the corresponding mode.

We found that the effective decay rate of the survival probability still remains as the overlap integral of the measurement-induced level-broadening function and the interacting spectral distribution. In conclusion, *without projection measurements*, we can observe both the QAZE and the QZE by means of quasi-measurements.

Moreover, we derived a correction to the previously known QZE/QAZE decay rate due to repopulation of the initial level. We obtained a quantitative criterion for the weakness of the repopulation effect and showed that repopulation can be neglected for a weak atom-reservoir coupling (when the free evolution of the initial level is exponential), but cannot be neglected for the case of a strong coupling.

Generally speaking, the QZE and QAZE stem from frequent decoherence events, which destroy the off-diagonal density matrix elements. When the diagonal elements in the density matrix remain unchanged after these processes, the above decoherence is actually dephasing between the initial and final states, e.g., between the first and second terms on the right hand side of equation (5). And the model in this paper is precisely of this type. Other methods include measurements as in Refs. 26,27,28,39,41 and even a classical random field^42^. Note that the above decoherence can take effect due to not only dephasing, but also a destruction of the final states^40^,^43^. On the other hand, the decoherence can be suppressed by a train of ultrafast off-resonant optical pulses^44^_._

We mention also a dynamic treatment of the QZE and the QAZE in a solid-state system^45^,^46^_. _This work^45^,^46^ significantly differs from the present paper in several respects, including the free evolution of the system, the measurement scheme, and the theoretical treatment. In particular, in Refs. 45, 46 the measurements are performed due to a static, non-resonant Coulomb interaction, which differs both physically and formally from the quasi-measurements by resonant laser pulses considered here.

## Methods

### Time evolution in spontaneous decay

We present the detailed calculations for the free evolution. We can integrate equation (6b) to have a formal solution for *β_k_*(*t*), i.e., 
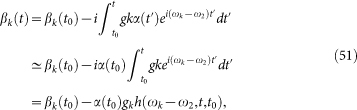
where in the second line we have used the short-time approximation 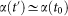 and *h*(*ω*,*t*,*t*_0_) is given by equation (8). By substituting equation (51) into equation (6a) and making use of the short-time approximation, we have 
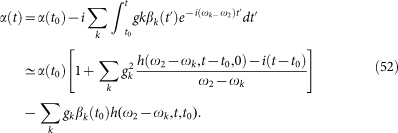


### Derivation of equations (35) and (36)

The quantity *α^(j,m)^* is given by equation (7a) with *t* = *τ_j_*_−1_ + *τ*, *t*_0_ = *τ_j_*_−1_, and 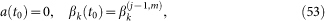
where 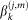 is the amplitude of the *k*th mode at the end of the *j*th cycle if the excitation leaves the atom in the *m*th cycle. Using equation (7b) in the interval (*τ_m_*, *τ_j_*) with the initial conditions 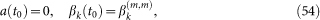
and in the interval (*τ_m_*_−1_, *τ_m_*_−1_ + *τ*) with the initial conditions 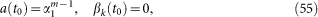
we obtain that 
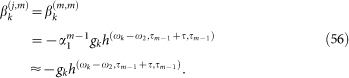
On using equation (56) in equation (53), we obtain from equation (7a) that 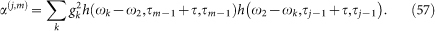
Then, using equations (31) and (32) and the equality 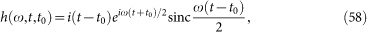
we recast equation (57) in the form 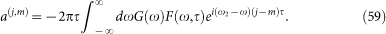
This equality implies equations (35) and (36).

## Author Contributions

Q.A. and A.K. wrote the main manuscript text, Q.A. and D.Z.X. did the calculations, S.Y., C.P.S. and F.N. designed the project. All authors reviewed the manuscript.
